# Composition, characteristics, and treatment technologies of condensable particulate matter present in flue gas emitted by coking plants in China

**DOI:** 10.1038/s41598-024-59098-0

**Published:** 2024-04-12

**Authors:** Chunyan Wang, Yuhong Du, Bo Yan, Yonggang Dong, Zhihui Zhao, Jinchao Shen, Mengxia Guo, Zhaochi Zhang

**Affiliations:** 1 Hebi Institute of Engneering and Technology, Henan Polytechnic University, Hebi, 458000 Henan People’s Republic of China; 2 College of Marine and Environmental Sciences, Tianjin University of Science and Technology, Tianjin, 300457 People’s Republic of China; 3 Henan Ecological Environment Monitoring Center, Zhengzhou, 450000 Henan People’s Republic of China

**Keywords:** Filterable particulate matter, Condensable particulate matter, Total particulate matter, Coking plant, Environmental sciences, Chemistry

## Abstract

To study the total particulate matter (TPM) in flue gas emitted by coking plants, a sampling system that could be used to collect filterable particulate matter (FPM) and condensable particulate matter (CPM) was designed and developed based on Method 202 recommended by the U.S. Environmental Protection Agency in 2017 and HJ 836-2017 issued by China. Using this system, FPM and CPM in flue gas emitted by four coking furnaces named A, B, C, and D were tested in China. Further, 9 water-soluble ions, 20 elements, and organic matter present in the CPM were simultaneously examined to determine their formation mechanisms. Statistical data suggested that the FPM emission level in the coking flue gas was low and the average mass concentration was less than 10 mg/m^3^. However, with high CPM and TPM emission levels, the TPM mass concentrations of A, B, C, and D were 130 ± 11.1, 84.4 ± 6.36, 35.1 ± 17.0, and 63.8 ± 13.0 mg/m^3^, respectively. The main component of TPM was CPM, and the average mass concentration of CPM accounted for 98%, 95%, 68%, and 95% of TPM in furnaces A, B, C, and D, respectively. Water-soluble ions were the important components of CPM, and the total concentration of water-soluble ions accounted for 70%, 87%, 42%, and 66% of CPM in furnaces A, B, C, and D, respectively. Toxic and harmful heavy metals, such as Mn, Cr, Ni, Cu, Zn, As, Cd, and Pb, were detected in CPM. The formation mechanism of CPM was analyzed in combination with flue-gas treatment. It was shown that the treatment process *“activated carbon– flue-gas countercurrent-integrated purification technology* + *ammonia spraying”* used in furnaces A and B was less effective in removing CPM, water-soluble ions, metals, and compounds than that of *“selective catalytic reduction denitrification* + *limestone–gypsum wet desulfurization (spraying NaOH solution)”* in furnaces C and D. Hence, different flue-gas treatment technologies and operation levels played vital roles in the formation, transformation, and removal of CPM from flue-gas. Organic components in CPM discharged from furnace A were determined via gas chromatography–mass spectrometry, and the top 15 organic components in CPM were obtained using the area normalization method. N-alkanes accounted for the highest proportion, followed by esters and phenols, and most of them were toxic and harmful to humans and ecosystems. Therefore, advanced CPM treatment technologies should be developed to reduce atmospheric PM_2.5_ and its precursors to improve ambient air quality in China.

## Introduction

The iron and steel industry has been an important source of atmospheric industrial emissions, and the soot emissions of the industry accounted for 29% of the total industrial particulate emissions^1^. Coking plants were the primary pollutant sources of the iron and steel industry, and the flue gas emitted by these plants contained complex components and multiple toxic and harmful substances^2^. According to the U.S. Environmental Protection Agency (EPA), primary particulate emissions from stationary sources include filterable particulate matter (FPM) and condensable particulate matter (CPM), and the addition of the two is termed total particulate matter (TPM). Solid and liquid particulate matter that can be captured by a filter in a test, known as FPM, stay suspended in emissions resulting from combustion, synthesis, decomposition of fuels and other substances, and mechanical handling of various materials. Such substances are called CPM, which are gaseous at the sampling location at the flue temperature and can be condensed into a liquid or solid state within a few seconds of cooling under environmental conditions after leaving the flue. CPM transform into particulate matter in the atmosphere with an aerodynamic equivalent diameter below 1 µm, which is an important precursor of fine particulate matter (PM_2.5_) and aerosol in the ambient air as well as an important component of haze formed under certain specific meteorological conditions^3^.

The current standard method for monitoring particulate matter in flue gas emitted by stationary sources in China was FPM, excluding CPM. Previous investigations on CPM emitted by stationary sources were not sufficiently comprehensive because their primary focus was on coal-fired power plants or boilers. Just a few studies concentrated on CPM found in flue gas emitted by waste incineration power plants, brick factories, electric arc furnaces, metallurgical plants, and other enterprises. The results showed that the CPM emission levels of enterprises in different industries differed greatly. The average CPM concentration of ultra-low emission from coal-fired power plants was generally less than 10 mg/m^3^^4^; however, the CPM concentration ranges of waste incineration power plants and building ceramic enterprises were 24.93–28.73^5^ and 9.08–102.12 mg/m^3^^6^, respectively. Generally, it was agreed that the emission levels of CPM and its contribution to atmospheric PM_2.5_ pollution could not be ignored^3–14^. Components of CPM emitted by different plants vary greatly because of different combustion sources, manufacturing techniques, and flue-gas treatment technology^4–7,14^.

Yang et al.^15^ measured the level of CPM emitted by the coking and sintering processes of iron and steel plants and reported that the mass concentrations of FPM_2.5_ (FPM with an aerodynamic equivalent diameter below 2.5 µm) and CPM discharged from coke ovens were 0.37 and 89.7 mg/m^3^, respectively, indicating that the CPM emission level was considerably higher than that of FPM_2.5_.

To further clarify the emission level of FPM, CPM, and TPM in flue gas emitted by coking plants in China, their mass concentrations were tested using a self-designed TPM sampling train, which could simultaneously collect FPM and CPM samples. Water-soluble ions, elements, and organic matter in CPM were tested simultaneously to analyze the formation mechanism and compare the treatment effect on pollutants by combining the existing flue-gas treatment technology units, thereby providing a technical basis for air pollution control.

## Materials and methods

### Sampling process

Four stable coking furnaces—A, B, C, and D—of coking plants in North China were randomly selected. The designed production capacity of coking furnaces A and B was 1.5 million t a^−1^, and the flue gas was discharged after implementing *“activated carbon–flue-gas countercurrent-integrated purification technology* + *ammonia injection.”* The designed production capacity of coking furnaces C and D was 700,000 and 600,000 t a^−1^, respectively, and the flue gas was discharged after being treated via *“SCR denitrification* + *limestone–gypsum wet desulphurization (spraying NaOH solution)”.* Table 1 lists the production conditions maintained during the test. Presampling preparation, sample collection, nitrogen blowing, and sample recovery were performed as per Method 202 recommended by EPA^16^. The constant velocity sampling method was employed, and the particulate matter was collected using a quartz filter (47 mm diameter), which was first dried in an oven for 4–5 h at a temperature above 300 °C and then placed in an anhydrous calcium sulfate dryer before the test. Sample weighing was performed at a temperature of 20 °C–23 °C and at a relative humidity of 30%–40%. To obtain true and overall data reflecting average concentrations of pollutants, five to six samples were collected from each stationary source; the volume of each sample was not less than 1 m^3^.

**Table 1:** Flue gas treatment process and production conditions maintained at the coking furnaces A, B, C, and D during the test.

Name of coking furnaces	The designed production capacity/(ten thousand t a^−1^)	Operating load/%	Flue gas treatment technology	Temperature of flue gas in stack/°C	Flue gas emission rate/(ten thousand m^3^ h^−1^)
A	150	86	Activated carbon—flue gas countercurrent integrated purification technology + spray ammonia	159	18
B	150	86		110	18
C	70	100	selective catalytic reduction (*SCR*)denitrification + wet limestone-gypsum desulfurization (spraying NaOH solution)	80	16
D	60	100		120	14

### Sampling equipment and methods

**Figure 1:**
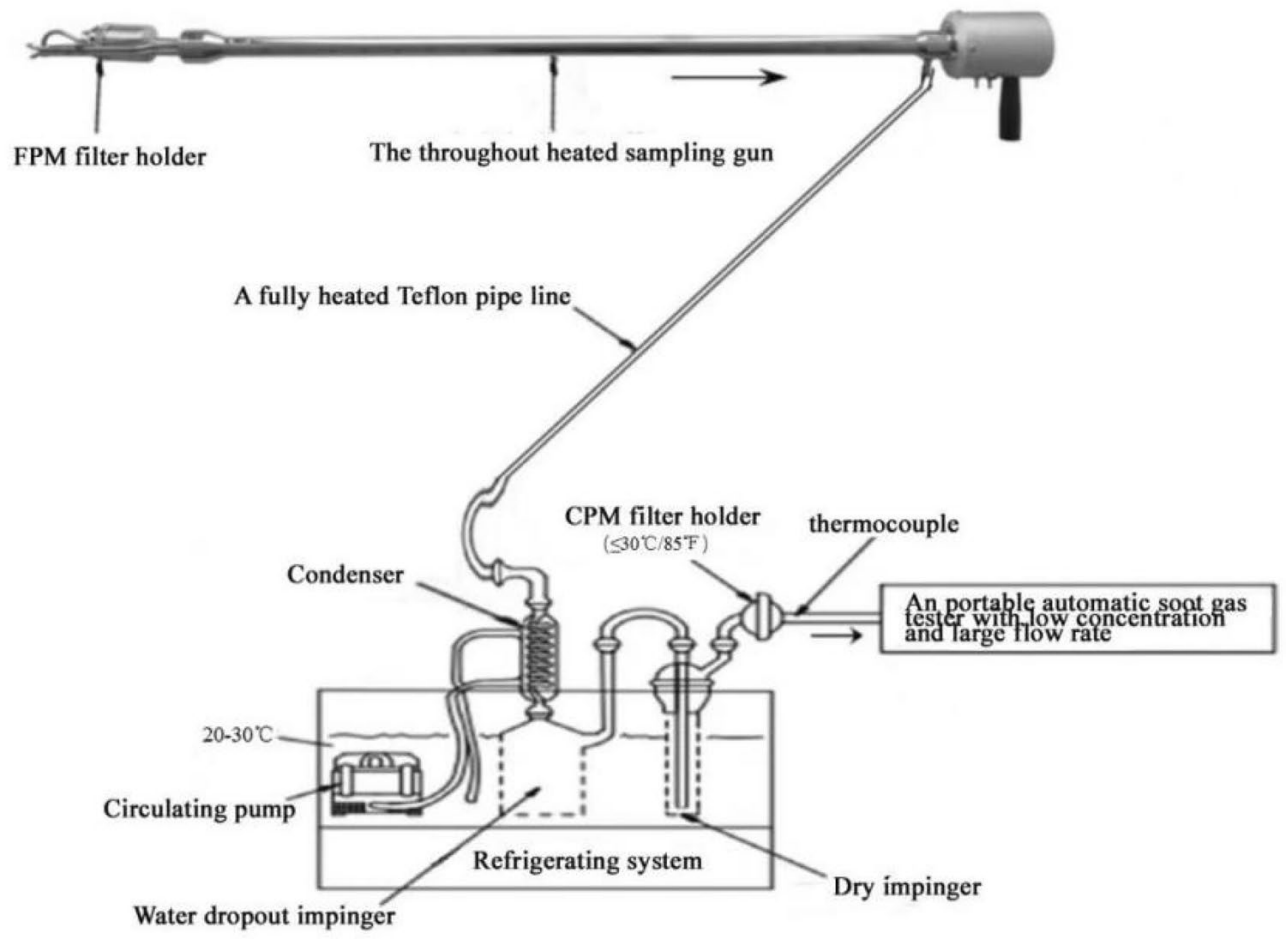
TPM Sampling system.

A sampling system, along with Method 202 (dry impinger method for determining condensable particulate emissions from stationary sources) and HJ836-2017(stationary source emission-determination of mass concentration of particulate matter at low concentrations-manual gravimetric method) issued by the Ministry of Environmental Protection of China, was employed to collect CPM emitted by a stationary source, which enabled the simultaneous collection of FPM and CPM samples. The system comprised FPM, CPM sample collection devices, and a smoke tester, which was specially used to collect particulate matter from flue gas emitted by stationary sources. In the sampling train, the smoke tester provided the energy for the entire system and could test the needed parameters, such as flue-gas temperature, flow rate, and sampling volume. Figure 1 shows the schematic of the system. The FPM part of the sampling system adopted in-stack filtration to meet the requirements of HJ 836-2017, and the CPM part of the sampling system met the requirements of Method 202. The front of the sampling gun was equipped with an FPM filter holder, and a filter was used to collect the FPM sample. The sampling gun behind the FPM filter holder was heated to a temperature of 110 °C–120 °C to prevent the condensation of water vapor and CPM. The vertically mounted condenser and water dropout impinger were connected by a completely heated Teflon pipeline, followed by a dry impinger, CPM filter holder, thermocouple, and portable automatic soot gas tester. The condenser cooled the flue gas to a temperature below 30 °C, and the condensate collection and dry impact bottles were placed in the thermostatic water tank maintained at a temperature of 20 °C–30 °C to ensure that the temperature of samples after passing through the CPM filter was within the range of 20 °C–30 °C.

### Sample analysis

#### FPM samples

As per HJ 836-2017, the mass concentration of FPM was determined using the gravimetric method.

#### CPM samples

Drop water and the CPM filter were used to collect CPM samples from the particulate matter newly formed on site. The mass concentration of CPM was determined using the gravimetric method. According to Method 202^16^, inorganic CPM samples could be collected. To simultaneously test the water-soluble ions and metal elements of inorganic CPM, inorganic CPM samples were primarily divided into three parts.

#### Inorganic CPM

First, sufficient ultra-filtered water (10 mL) was added to cover the CPM filter and placed in a clean extraction container. The extraction container was then placed in an ultrasonic bath for at least 2 min to extract the water-soluble substances. The extraction process was repeated three times. Then, the extracted water was combined with the drop water collected on site to form inorganic CPM samples while maintaining the total volume at 500 mL. Finally, the inorganic CPM sample was primarily divided into three parts for testing. The sample (250 or 300 mL) was transferred to a beaker not more than 500 mL in capacity and evaporated to a volume below 10 mL at 105 °C ± 1 °C followed by drying at room temperature (below 30 °C). After evaporation, the sample was dried in an anhydrous calcium sulfate dryer for 24 h and weighed every 6 h to ensure that its weight remained constant (the weight difference had to be not more than 0.5 mg). Two 50-mL inorganic CPM samples were then removed to test their water-soluble ions and metal elements. Tables 2 and 3 list the materials, reagents, and instruments used as well as the measurements taken during the test.

**Table 2:** Measurements and instruments used in the test.

Number	Factors of determination	Methods of determination	Instruments and equipment
1	F^−^, Cl^−^, SO_4_^2−^, NO_3_^−^, NH_4_^+^, Na^+^, K^+^, Mg^2+^, Ca^2+^	Ion chromatography	Ion chromatograph, ICS1500
2	Be, Se, Al, V, Ag, Cr, Mn, Sb, Fe, Mo, Co, Ni, Cu, Zn, As, Cd, Ba, Ti, Pb	Inductively coupled plasma mass spectrometry	Inductively coupled plasma source mass spectrometer, 7700X
3	FPM, Organic CPM_,_ Inorganic CPM	Gravimetric method	Precision electronic balance, CPA225D
4	Hg	Cold atomic absorption spectrophotometry	Cold atomic absorption mercury meter, LEEMAN LABS
5	Qualitative analysis of organic CPM	Gas chromatography-mass spectrometry	The gas chromatograph-mass spectrometer, Produced by Agilent in the United States, 8890-5977B

**Table 3:** Reagents and matierals.

Number	Reagents and materials	Grade or specification
1	Acetone	Superpure
2	n-hexane	Superpure
3	Carbinol	Superpure
4	Quartz filter membrane	Φ47 mm
5	Filter membrane	0.22 µm

#### Organic CPM

The mass concentration of organic CPM was determined as per Method 202 ^16^. To determine the organic components in CPM, organic CPM samples were collected and qualitatively analyzed via gas chromatography–mass spectrometry (GC–MS). The type of chromatographic column was HP-3. High-purity nitrogen (99.99%) was adopted as the carrier gas. The column temperature was 50 °C–270 °C, with a programmed temperature increase of 5 °C/min. The split ratio was 1:1, and the solvent delay time was 3 min. The organic CPM samples collected on site were processed as follows: First, the samples were placed in a centrifugal tube and their volumes were reduced to < 1 mL using a multifunctional nitrogen blower. Then, to improve detection sensitivity, the interfering components in the samples were removed via solid phase extraction, thereby obtaining pure concentrated liquid samples. Subsequently, the purified liquid samples were concentrated to near dry by blown with nitrogen. Thereafter, methanol, acetone, and n-hexane were added to a centrifuge tube at a constant volume of 1 mL. Finally, the mixture was inhaled using a 1-mL needle tube and then poured into the receiving bottle using a 0.22-µm filter membrane for qualitative analysis.

### Quality control measures

The quality of all types of reagents and ultra-filtered water used in the laboratory analysis met the requirements of analytical methods. The samples collected on site were sent to the laboratory and analyzed as soon as possible. The balance should be used within the validity period of verification, and its performance should be maintained during verification. The weighing operation was conducted at a temperature of 20 °C–23 °C and relative humidity of 30%–40%, and the weighing was the same balance and personnel. The balance was calibrated before use to avoid the influence of static electricity. The correlation coefficient of the standard curve used during the water-soluble-ion analysis was not < 0.995. The internal standard method was used to check whether the instrument signal was drifting or interfered, i.e., for each batch of samples (no more than 20 samples), a standard solution with the concentration of the intermediate point of the standard curve should be measured and the relative error between the determination results and the concentration of the point should be less than 10%. For each batch of samples (no more than 20 samples), the blank sample, biparallel sample, and recovery rate were determined. The relative deviation of parallel samples was < 20%, and the recovery rate was between 80 and 120%. Calibration curves were drawn for each analysis, and the correlation coefficient exceeded 0.999. Other quality control measures adopted for the element analysis were the same as those used during the water-soluble-ion analysis. Before the qualitative analysis of organic CPM, its GC–MS performance was verified to ensure that it met the test requirements and a blank sample was used in advance to avoid interference from organic CPM samples.

## Results and discussion

### Emission levels and characteristics of FPM, CPM, and TPM

The measurement results of FPM, CPM, and TPM are shown in Table 4. The level of FPM emission of the four coking furnaces was low with an average mass concentration of not more than 10 mg/m^3^, indicating that the FPM removal from flue-gas treatment facilities was effective. Moreover, the levels of CPM and TPM emissions of the four coking furnaces were higher than that of FPM. The average mass concentrations of FPM, CPM, and TPM considerably varied because of the differences in the flue-gas treatment technologies used and their operation levels. Coking furnaces A and B used the treatment process *“activated carbon–flue-gas countercurrent-integrated purification technology* + *ammonia injection,”* in which activated carbon was used to adsorb SO_2_, NOx, dioxins, heavy metals, and other pollutants in the flue gas, and ammonia water, a reducing agent, was sprayed to remove NOx. The treatment process *“SCR denitrification* + *limestone–gypsum wet desulphurization (spraying NaOH solution)”* was used in furnaces C and D, which employed SCR denitrification to remove NOx and the limestone–gypsum method to remove sulfur oxides. Both CPM and TPM emission levels of furnaces A and B were considerably higher than those of furnaces C and D, indicating that the treatment process *“SCR denitrification* + *limestone–gypsum wet desulfurization (spraying NaOH solution)”* had a more effective CPM treatment than the treatment process *“activated carbon–flue-gas countercurrent-integrated purification technology* + *spraying ammonia.”* This was because of the synergistic effect of the limestone–gypsum desulfurization on FPM and CPM^17^. The mass concentrations of TPM emitted by furnaces A, B, C, and D were 130 ± 11.1, 84.4 ± 6.36, 35.1 ± 17.0, and 63.8 ± 13.0 mg/m^3^, respectively, and the corresponding average percentages of CPM in TPM were 98%, 95%, 68%, and 95%, respectively, indicating that CPM was the main component of TPM. According to the definition of CPM^16^, CPM is a liquid or granular substance formed by the condensation of flue gas emitted into the atmosphere due to the decrease in ambient temperature. Using certain flue-gas treatment technologies and under operating conditions, FPM can be converted into CPM when the flue-gas temperature increases^8^. The emission temperatures of flue gas emitted by coking furnaces A, B, C, and D were 159 °C, 110 °C, 80 °C, and 120 °C, respectively, which are much higher than the CPM sample collection temperatures of 20 °C–30 °C. During sampling, first, the flue gas entered the sampling gun and was filtered using an FPM filter membrane. Next, it was condensed by a condenser, which maintained the temperature between 20 and 30 °C by circulating water. Thus, the CPM substance in flue gas was condensed and filtered using a CPM filter to form a CPM sample. Hence, the higher emission temperature of flue gas emitted by the coking was the critical reason for the higher CPM content. In this study, the flue-gas temperature of furnace C was the lowest and part of the CPM was converted to FPM, which could be removed by the particulate matter removal process. This was also proved by the low mass concentration of CPM and TPM emissions.

**Table 4:** Mass concentrations of FPM, CPM and TPM emitted by coking furnaces A, B, C, and D.

Name of Coking furnace	Mass Concentration	FPM	Organic CPM	Inorganic CPM	CPM	TPM	CPM/TPM
A	(C ± S)/( mg m^−3^)	2.65 ± 0.73	2.98 ± 0.80	124 ± 10.1	127 ± 10.4	130 ± 11.1	0.98 ± 0.93
	RS/%	28	27	8	8	36	23
B	(C ± S)/( mg m^−3^)	3.82 ± 0.74	1.75 ± 0.57	79.1 ± 5.78	80.6 ± 5.62	84.4 ± 6.36	0.95 ± 0.88
	RS/%	19	33	7	7	26	27
C	(C ± S)/( mg m^−3^)	9.95 ± 4.05	3.84 ± 2.06	21.3 ± 13.1	25.1 ± 14.1	35.1 ± 17.0	0.68 ± 0.13
	RS/%	41	54	62	56	48	20
D	(C ± S)/( mg m^−3^)	3.25 ± 1.24	5.93 ± 0.69	54.6 ± 11.4	60.5 ± 11.8	63.8 ± 13.0	0.95 ± 0.01
	RS/%	38	12	21	20	20	1

C ± S” stand for mean ± standard deviation, “RS” stand for relative standard deviation, and the following was the same.

Coking plants in China that employed the flue-gas treatment technology presently had higher CPM and TPM emission levels than the coal-fired power plants^10^ and waste incineration power plants ^18^. The primary component of TPM in the flue gas emitted by furnaces A, B, C, and D was CPM, and the corresponding emission levels of TPM were 48, 21, 3, and 19 times the emission levels of FPM, respectively. CPM accounted for > 95% of TPM in the flue gas emitted by furnaces A, B, and D. Because of the lower mass concentration of CPM and the higher concentration of FPM, CPM accounted for 68% of TPM in the flue gas emitted by furnace C. It was noted that 98%, 95%, 68%, and 95% of primary particulate matter in the emissions of coking furnaces A, B, C, and D, respectively, had not been measured because of the incomplete testing methods, which resulted in greatly underestimated levels of primary particulate matter emitted by stationary sources. If the CPM emission factor of the stationary source was considered, the contributions of particulate matter emitted by industrial combustion sources in Chungcheongnam-do and Jeollanam-do of South Korea to regional particulate matter pollution increased from 87.3% and 78.7% to 94.5% and 96.5% respectively^19^. Once the CPM emitted by stationary sources entered into the atmosphere, due to the reduction of environmental temperature, “heterogeneous nucleation” or “homogeneous nucleation” would occur, thus forming PM_2.5_^20^. Therefore, strict control of CPM in flue-gas is an important measure for the emission reduction of atmospheric PM_2.5_ and its precursors and is of great significance to further improve environmental air quality.

CPM emitted by furnaces A, B, C, and D primarily comprised inorganic matter; the corresponding average mass concentration of inorganic matter accounted for 98%, 98%, 85%, and 90% of the CPM, respectively. The organic content of CPM was small with an average mass concentration below 6 mg/m^3^. These results agreed with the conclusions made by other scholars^14,21,22^.

### Composition and characteristics of CPM

#### Characteristics of water-soluble ions in CPM

**Figure 2:**
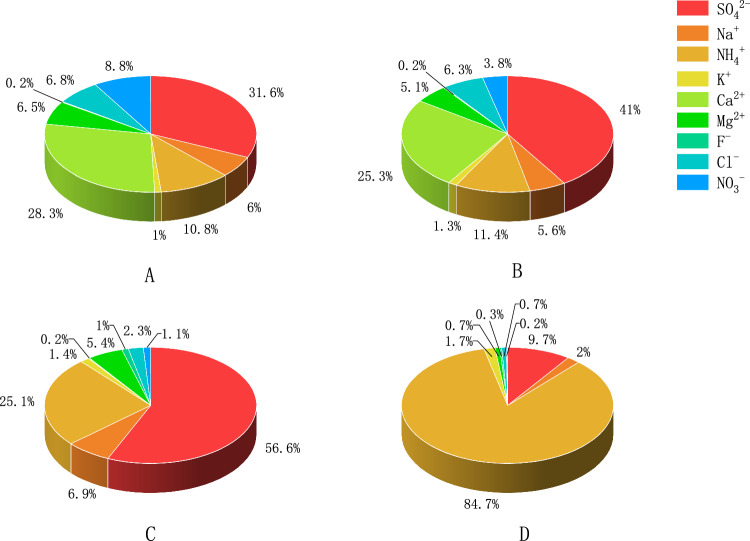
Mass concentration percentage of each water-soluble ion in the total weight of CPM emitted by the coking furnaces A, B, C, and D.

Table 5 lists the mass concentrations of water-soluble ions in CPM emitted by furnaces A, B, C, and D, and the mass concentration percentages of each type of water-soluble ions in CPM are shown in Fig. 2. The total mass concentration of water-soluble ions discharged from furnaces A, B, C, and D were 88.4 ± 28.6, 70.0 ± 6.21, 10.5 ± 6.45, and 42.3 ± 9.83 mg/m^3^, respectively, accounting for 70%, 87%, 42%, and 66% of the CPM, respectively. Thus, water-soluble ions were important components of CPM. Compared with coal-fired power plants^10^ and waste incineration power plants^18^, coking plants had a higher total mass concentration of water-soluble ions, leading to higher CPM emission levels.

**Table 5:** Water-soluble ion mass concentrations in CPM emitted by the coking furnaces A, B, C and D.

Name of Coking furnace	Mass Concentration	Na^+^	NH_4_^+^	K^+^	Ca^2+^	Mg^2+^	F^-^	Cl^-^	NO_3_^-^	SO_4_^2-^	Total Weight
A	(C ± S)/(mg m^−3^)	5.33 ± 1.13	9.56 ± 3.00	0.90 ± 0.51	25.0 ± 5.63	5.76 ± 1.32	0.15 ± 0.03	5.98 ± 1.45	7.77 ± 10.9	27.9 ± 6.81	88.4 ± 28.6
	RS/%	21	31	57	20	23	20	24	140	24	32
B	(C ± S)/(mg m^−3^)	3.95 ± 0.54	7.95 ± 3.27	0.88 ± 0.07	17.7 ± 1.43	3.58 ± 1.08	0.12 ± 0.01	4.44 ± 0.34	2.64 ± 0.66	28.7 ± 3.45	70.0 ± 6.21
	RS/%	14	41	8	8	30	8	8	25	12	9
C	(C ± S)/(mg m^−3^)	0.72 ± 0.17	2.63 ± 1.44	0.15 ± 0.14	0.02 ± 0.02	0.56 ± 0.50	0.10 ± 0.10	0.24 ± 0.08	0.12 ± 0.05	5.92 ± 4.42	10.5 ± 6.45
	RS/%	24	55	94	105	91	94	34	44	75	62
D	(C ± S)/(mg m^−3^)	0.83 ± 1.10	35.9 ± 4.95	0.71 ± 1.39	0.01 ± 0.01	0.28 ± 0.24	0.13 ± 0.20	0.31 ± 0.28	0.08 ± 0.02	4.10 ± 3.45	42.3 ± 9.83
	RS/%	24	55	94	98	91	94	34	44	75	62

The CPM emissions of furnaces A and B had a high content of water-soluble ions and almost the same composition characteristics. SO_4_^2−^ and Ca^2+^ in CPM had the highest mass concentrations, followed by NH_4_^+^, NO_3_^−^, Cl^−^, Na^+^, and Mg^2+^. These seven ions accounted for > 98% of the total weight of water-soluble ions in the CPM, whereas the mass concentrations of K^+^ and F^−^ were the lowest, accounting for even < 2%.

Flue gas produced by the coking furnaces contained complex pollutants. The main pollutants were soot, SO_2_, and NOx, and the flue-gas treatment process was primarily dust removal, desulfurization, and denitrification. SO_2_ in the flue gas could be further oxidized to SO_3_ during the treatment process, and SO_4_^2−^ was produced in the presence of H_2_O^9^. During denitrification, NH_3_∙H_2_O was used as the reducing agent. NH_3_ could react with NOx to form N_2_ and H_2_O. However, because the flue gas produced in the coking process was dynamic and complex, the amount of NH_3_∙H_2_O injection was difficult to accurately calculate. During the denitrification process, NH_3_∙H_2_O was usually excessively injected. The excessive NH_3_∙H_2_O and ammonia that failed to participate in the denitrification reaction resulted in ammonia escape and formed NH_4_^+^ in the presence of acidic gases, such as SO_2_ and NOx, in the flue-gas. In the presence of SO_2_ and H_2_O, NH_3_ would also react adversely on the catalyst surface to form (NH_4_)_2_SO_4_ and NH_4_HSO_4_^18^. These were the crucial formation mechanisms of SO_4_^2−^ and NH_4_^+^ in CPM.

As mentioned previously, the treatment process *“activated carbon–flue-gas countercurrent-integrated purification technology* + *ammonia injection”* used in furnaces A and B has little effect on CPM removal. The mass concentrations of SO_4_^2−^ in CPM emissions of furnaces A and B, which were 27.9 ± 6.81 and 28.7 ± 3.45 mg/m^3^ respectively, were considerably higher than that of furnace C or D, which were 5.92 ± 4.42 and 4.10 ± 3.45 mg/m^3^ respectively. Thus, the treatment process *“activated carbon–flue-gas countercurrent-integrated purification technology* + *ammonia spraying”* employed in furnaces A and B was less effective in removing SO_2_ compared with the treatment process *“SCR denitrification* + *limestone–gypsum wet desulfurization(spraying NaOH solution)”* employed in furnaces C and D. Ca^2+^, NO_3_^−^, Cl^−^, Na^+^, and Mg^2+^ had relatively high mass concentrations in CPM emitted by furnaces A and B, and the mass concentrations of Ca^2+^ were as high as 25.0 ± 5.63 and 17.7 ± 1.43 mg/m^3^, respectively. These ions primarily came from the coking process of coal and blast furnace gas as the energy source. However, considering the CPM of furnaces C and D, the ion concentrations were greatly reduced and their mass concentrations were less than 1 mg/m^3^. The limestone–gypsum wet desulfurization process was a good synergistic treatment of pollutants.

The water-soluble levels in CPM emissions of furnaces C and D were low, and the composition characteristics of water-soluble ions were consistent. The mass concentrations of SO_4_^2-^ and NH_4_^+^ were the highest, and the sum of the average mass concentrations of these two ions accounted for 81% and 95% of the total mass concentration of water-soluble ions of furnaces C and D, respectively; however, the mass concentrations of other ions were < 1 mg/m^3^. The content of NH_4_^+^ in CPM emissions of furnace D was high (35.9 ± 4.95 mg/m^3^), which was considerably higher than that of furnace C (2.63 ± 1.44 mg/m^3^), indicating excessive ammonia injection in furnace D during denitrification. Thus, the amount of NH_3_∙H_2_O injected should be controlled precisely to avoid the formation of NH_4_^+^ to realize a lower level of CPM emission.

In summary, owing to the different flue-gas treatment processes adopted by the four coking furnaces, the contents of water-soluble ions in CPM were considerably different, which directly affected the emission levels of CPM and TPM. Compared with limestone–gypsum wet desulphurization in furnaces C and D, the CPM discharged by activated carbon desulphurization in furnaces A and B contained higher concentrations of SO_4_^2−^. Moreover, limestone–gypsum wet desulphurization had better synergic treatment of other pollutants and, hence, was a better desulfurization process. In addition, the excessively high concentration of NH_4_^+^ in CPM discharged from furnace C indicated that the operating conditions and levels of flue-gas treatment facilities were crucial factors affecting CPM emission. Hence, an accurate and reasonable ammonia injection quantity was an important condition for reducing NH_4_^+^ concentration in CPM.

#### Characteristics of metal elements in inorganic CPM

Table 6 lists the mass concentrations of metal elements (≥ 0.001 µg/m^3^) in inorganic CPM emitted by the four coking furnaces. The total concentrations of the elements in inorganic CPM emitted by furnaces A, B, C, and D were 74.6 ± 5.31, 63.0 ± 12.3, 10.5 ± 12.2 and 7.34 ± 4.12 µg/m^3^, respectively. Because furnaces A and B or furnaces C and D used the same flue-gas treatment technology, the levels of the total mass concentration of elements in the inorganic CPM were consistent. However, the total elemental mass concentrations in emissions of furnaces A and B were considerably higher than that of furnaces C and D, indicating that the treatment process *“activated carbon–flue-gas countercurrent-integrated purification technology* + *ammonia spraying”* employed in furnaces A and B had less effect on the removal of metal elements and their compounds in inorganic CPM than the treatment process *“SCR* + *limestone–gypsum wet desulfurization(spraying NaOH solution)”* employed in furnaces C and D. The mass concentrations of Ba and Al in CPM emitted by furnaces A and B were high: 39.1 ± 3.12 and 29.0 ± 4.30 µg/m^3^, respectively, in the case of furnace A and 34.4 ± 5.88 and 23.0 ± 4.15 µg/m^3^, respectively, in the case of furnace B, accounting for 91% and 91% of the total elemental mass concentrations, respectively. However, the mass concentrations of other metal elements were low.

**Table 6:** Mass concentrations of elements (µg·m^-3^) in the inorganic CPM emissions of the furnaces A, B, C and D.

Name of elements	A		B		C		D	
	Mass concentration		Mass concentration		Mass concentration		Mass concentration	
	C ± S	RS/%	C ± S	RS/%	C ± S	RS/%	C ± S	RS/%
Al	29.0 ± 4.30	20	23.0 ± 4.15	18	1.65 ± 2.72	164	−	–
V	0.30 ± 0.01	5	0.24 ± 0.02	8	0.01 ± 0.01	100	0.04 ± 0.02	64
Cr	0.09 ± 0.01	6	0.07 ± 0.01	8	0.05 ± 0.01	25	0.06 ± 0.05	79
Mn	0.38 ± 0.06	16	0.32 ± 0.06	18	0.77 ± 0.93	122	0.01 ± 0.01	245
Fe	0.05 ± 0.11	220	0.19 ± 0.34	180	0.44 ± 1.09	245	–	−
Co	0.02 ± 0.00	0	0.02 ± 0.00	0	0.01 ± 0.01	88	0.02 ± 0.02	95
Ni	0.17 ± 0.03	16	0.14 ± 0.02	10	0.32 ± 0.35	109	0.01 ± 0.02	237
Cu	0.21 ± 0.04	20	0.29 ± 0.15	52	0.09 ± 0.21	233	0.62 ± 0.28	46
Zn	0.78 ± 0.92	118	0.40 ± 0.28	70	3.11 ± 2.93	94	−	−
As	1.39 ± 2.15	155	0.78 ± 1.08	140	0.17 ± 0.10	63	0.17 ± 0.15	85
Se	0.48 ± 0.11	23	0.91 ± 0.16	18	1.30 ± 1.22	94	2.45 ± 1.60	65
Mo	1.92 ± 0.11	6	1.52 ± 0.14	9	1.46 ± 2.34	160	1.91 ± 3.24	170
Cd	0.001 ± 0.001	100	–	–	0.01 ± 0.01	84	0.03 ± 0.02	81
Sb	0.68 ± 0.05	8	0.64 ± 0.07	11	–	–	–	–
Ba	39.1 ± 3.12	8	34.4 ± 5.88	17	0.04 ± 0.04	99	0.07 ± 0.08	117
Tl	0.005 ± 0.005	100	0.002 ± 0.001	50	0.99 ± 0.89	90	1.97 ± 1.47	75
Pb	0.003 ± 0.006	200	0.007 ± 0.006	80	−	−	0.01 ± 0.01	57
Total weight	74.6 ± 5.31	7	63.0 ± 12.3	8	10.5 ± 12.2	116	7.34 ± 4.12	56

“-” stand for not being detected.

The heavy metals tested included Mn, Cr, Ni, Cu, Zn, As, Cd, and Pb. The levels of Cr in CPM emitted by furnaces A, B, C, and D were consistent. Emissions from furnace C had the highest levels of Mn, Ni, and Zn, and emissions from furnace D had the highest levels of Cu and Pb. The levels of As in the emissions of furnaces A and B were considerably higher than those in the emissions of furnaces C and D, whereas the levels of Cd in the emissions of furnaces C and D were higher than those in the emissions of furnaces A and B. The content of metals and their compounds in CPM emitted by the coking furnaces were related to both the flue-gas treatment technology and the quality of coal used in coking and blast furnace gas production.

PM_2.5_ in the atmosphere contains various harmful heavy metals such as Mn, Pb, Cu, Cd, Zn, Cr, and Ni^23,24^. Once heavy metals, such as Pb, As, and Cd, enter the atmosphere or organisms, their valence states change during their migration and transformation and accumulate via the food chain to endanger human health without getting decomposed^21^. Therefore, from the perspective of the source and sink, the metals and their compounds in CPM that entered the atmosphere were the source of metal elements in PM_2.5_ present in the atmosphere. Hence, the technologies used for monitoring CPM and its metals, especially heavy metals, and research conducted on the effects of CPM and its metals on the ecological environment should be strengthened to ensure the safety of the ecological environment in China.

#### Characteristics of organic CPM

**Figure 3:**
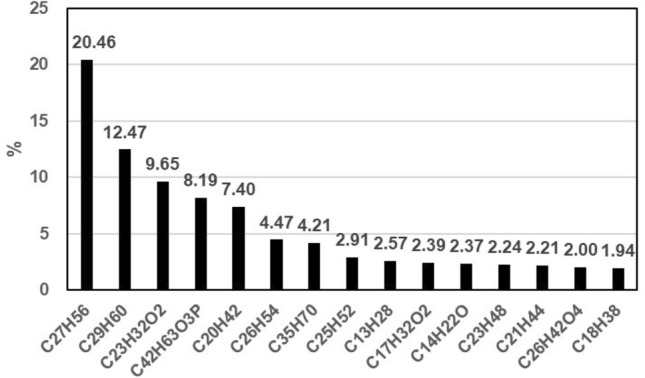
Percentage of the 15 main components of organic CPM.

Because of the limitations imposed by sampling conditions, organic CPM in flue gas emitted by coking furnace A was separately collected for qualitative analysis. Because organic compounds could be dissolved by different solvents, n-hexane, methanol, and acetone were selected as the final constant volume solvents, and the full-scan ion current diagram was obtained via GC–MS. The organic compounds tested in CPM, are shown in Table 7. Using the area normalization method, the percentage of the peak area of each component in the chromatogram to the peak area of the total substance was used to obtain the percentage of the 15 important organic components in the CPM, as shown in Fig. 3. The organic compounds represented by the formulas indicated from left to right on the X axis were n-heptacosane, n-doxane, 2,2′-methylene di-(4-methyl-6-tert-butylphenol), tri(2,4-di-tert-butylbenzene) phosphite, n-eicane, n-hexane, (Z)-35-carbon-17-ene, n-25ane, n-trisecane, methyl septanate, 2,4-di-tert-butyl basic phenol, n-docotriane, n- × 21, dinonyl phthalate, and n-octadecane. The figure demonstrates that the proportion of n-alkanes was the highest, followed by those of esters and phenols. Most organic substances in the atmosphere are toxic to humans and ecosystems.

**Table 7:** Organic components in the CPM.

Sorts of organic matter	organic components
Hydrocarbon	N-tridecane, n-tetradecane, n-pentadecane, n-cetane, n-heptadecane, n-eicosane, n-octadecane, n-tetracosane, n-pentaccosane, n-nonacosane, n-hentriacontane, n-tetratetracontane, 2,6,11,15-tetramethylhexadecane, 2,6,10-trimethyltetradecane, pristane, and (Z)-35-carbon -17-ene
Esters	Dimethyl phthalate, n-butyl-isooctyl phthalate, dioctyl phthalate, dioctyl carbonate, triethyl phosphate, butyl-chloride, glycerol tridodoalkate, tri (2,4-di-tert-butyl-butyl) phenyl phosphite, methyl septanate, methyl stearate, methyl stearate, methyl farnite
Phenols	2,4-di-tert-butylphenol, 2, 6-di-tert-butylhydroquinone, 2,2′-methylene bis -(4-methyl-6-tert-butylphenol), phenol
Alcohols	Dodecyl alcohol, hexadecyl alcohol, and octadecyl alcohol
acids	5,8,11, 14-eicosapotebutyric acid and eucenoic acid
Aromatic hydrocarbons	3,4′-diethyl-1,1′-biphenyl
Aldehydes	hydroxymethylfurfural
Ketone	1-hydroxycyclohexylbenzenone

To summarize, the emission level of FPM in the flue gas was low with an average mass concentration of < 10 mg/m^3^ and the emission level of CPM was high. CPM contains an abundant quantity of water-soluble ions as well as Cr, Ni, Cu, Zn, As, Cd, Pb, and other heavy metals, which are harmful to human health. Organic compositions, such as esters and phenols, had also not been neglected. Because CPM formed PM_2.5_ and its precursors in the atmosphere, the treatment of CPM in the flue gas was important to reduce the amounts of PM_2.5_ and its precursors present in the atmosphere, which was crucial to safeguarding human health and the ecosystem.

### Discussion on the treatment of CPM in the flue gas

#### Reduction of flue-gas temperature to remove CPM from the flue gas

According to the formation mechanism of CPM in flue gas, the temperature of flue gas at the chimney exit, the flue-gas treatment technology, and operation levels were the key factors of CPM formation and its content in flue gas. The following is a discussion of CPM governance techniques based on these factors.

When high-temperature flue gas was discharged in the atmosphere at a low ambient temperature via chimney exits, the gas chemically transformed into low volatile vapor, which underwent homogeneous nucleation or direct transformation into droplets in the accumulation mode (0.05 µm < diameter of particles < 2 µm)^25^. Thus, temperature was reported to be an important factor in determining the condensation of CPM to form fine particles. Therefore, reduction of flue-gas temperature could promote the condensation of gaseous CPM into liquid or fine particles and their conversion into FPM. The temperatures of the flue gas emitted by furnaces A, B, C, and D tested in this study were 159 °C, 110 °C, 80 °C, and 120 °C, respectively, which were considerably higher than the ambient temperature in air. Therefore, the cooling and condensation method could be used to transform gaseous CPM to FPM, and the advanced particle removal facilities could be employed thereafter to remove FPM. These measures would improve the CPM removal efficiency of existing flue-gas treatment facilities. Studies demonstrated that the low-temperature electric dust removal technology adopted by coal-fired power plants with ultra-low emissions could remove most of their SO_3_ emissions using a low-temperature economizer (MGGH) to reduce the temperature of flue gas from 130 °C–140 °C to 90 °C–100 °C, improving the dust removal effects of wet desulfurization and the efficiency of FPM and CPM removal^9^, which could enable the mass concentration of TPM to reach ~ 5 mg/m^3^^18^. If the flue-gas temperature dropped, the conversion of gaseous CPM to FPM could be increased, further improving the efficiency of TPM removal. Water-soluble ions, heavy metals, organic compounds, and other components in CPM would be dissolved or bound during its condensation. Therefore, the removal of CPM using a cooling and condensation process was a collaborative treatment for multiple pollutants. It was clear that exploiting low-cost cooling and condensation treatment technology was particularly important because its economic feasibility was the basis for widespread application. A multipollutant collaborative treatment system based on TPM removal in wet flue gas was devised^18^. This system was suitable for winter when the atmospheric ambient temperature was below 10 °C and air pollution occurred frequently. With external low-temperature air as the cold source as well as the designing of the induced draft fan, air distribution pipe, air distribution hole, and diversion defog mechanism, the air distribution hole was set between the upper and lower two adjacent diversion defog mechanisms. In addition, the system could realize the cooling and pressurization of flue gas by the outside air and increase the probability of collision and growth of particles to promote the condensation of water and gas compositions in the flue-gas. FPM and CPM in flue gas would dissolve or be trapped in droplets with its formation, expansion, and collision. The droplets would form condensate water under the action of gravity. Thus, CPM, including heavy metals and their compounds, organic matter, and gaseous pollutants such as SO_2_ and NOx, would dissolve in the droplets and be removed from the flue gas when the droplets fall. Thus, the collaborative treatment efficiency of TPM and other pollutants was higher, and the emission reduction effect was greatly improved. This process equipment was simple in structure, and it mainly functioned as a power pump, which has low construction and operation costs. After the high wet flue gas formed by the limestone–gypsum wet desulfurization process was cooled and condensed, part of FPM and CPM would be removed with the condensation of water and other emissions in the flue gas. In addition, part of SO_2_, NOx, and other acidic gases in flue gas would be dissolved in water and removed, and the condensed liquid with water as the main component was acidic. The content of pollutants in the condensed water was relatively low, and it could be used for greening irrigation or landscape water after simple treatment using the acid–base balance method. The moisture content in the flue gas of the activated carbon desulfurization process was low, CPM condensation was primarily conducted to form FPM, and the ash from the dust removal could be treated as solid waste.

#### Optimization of the existing flue-gas treatment process to improve the efficiency of CPM treatment

The optimization of the currently used flue-gas treatment process included two steps: optimization of the flue-gas treatment process with a high TPM removal efficiency and improvement of the operation levels of flue-gas treatment facilities. In this study, the treatment process “SCR denitrification + limestone–gypsum wet desulphurization + spraying NaOH solution” used in the tested flue gas had a better CPM treatment effect than the treatment process “activated carbon flue-gas countercurrent-integrated purification technology + spraying ammonia,” indicating that different flue-gas treatment processes produced different CPM emission levels. The wet desulfurization and wet electric dust removal devices used in coal-fired power plants could remove CPM and FPM synergistically at low levels of TPM emissions^17^. Therefore, the monitoring and evaluation of the effects caused by FPM and CPM removal should be strengthened using the flue-gas treatment technology presently used in China, and the processes and equipment used in FPM and CPM removal should be optimized to achieve the best TPM treatment effect. In this study, the mass concentration of NH_4_^+^ in CPM emitted by furnace D was high (35.9 ± 4.95 mg/m^3^) owing to excessive ammonia injection during NOx removal. It was much higher than in other emission sources. Therefore, the optimization of the flue-gas treatment process to improve the removal efficiency of NH_4_^+^ from CPM was extremely important.

#### Use of new nanofiltration materials in CPM treatment technologies

There has been a breakthrough in research on PM_2.5_ nanofiltration materials. A flexible electrostatic spinning organic/inorganic composite SiO_2_ nanofiber filtration membrane with a fine fiber diameter and uniform distribution by calcination at ~ 800 °C was prepared in the laboratory. Without folding, the filtration efficiency of the material for PM_2.5_ was 95.8%, and the resistance was ~ 40 Pa. When the fiber membrane was folded 90°, the filtration efficiency was 95.9% and the resistance was ~ 40 Pa. It was then folded 180°, with a filtration efficiency of 96.1% and a resistance of ~ 41 Pa. Using water, anhydrous ethanol, 84 disinfectants, and high-temperature calcination to treat the polluted fiber film, pollutants such as sodium chloride aerosol and incense fumes were removed and the filtration efficiency of the fiber film was unchanged. Hence, the material had high efficiency, low resistance, and satisfactory mechanical strength and structural stability and could remove adsorbed pollutants through cleaning or high-temperature calcination, thereby making it reusable^26^. Thus, this material had good prospects for use in CPM removal and its use would effectively enhance the existing flue-gas treatment processes to a new level.

## Conclusions

Through the samples collection of FPM and CPM from flue gas emitted by furnaces A, B, C, and D of coking plants in North China, the mass concentrations of FPM and CPM were tested. To clarify the composition, characteristics, and formation mechanism of CPM, the tests of water-soluble ions, element analysis and qualitative analysis of organic matter were performed. The findings of the analyses are as follows:

1. The level of FPM in the flue gas of the four coking furnaces was low, and the average mass concentration of FPM was < 10 mg/m^3^. Moreover, the levels of CPM and TPM emissions were high. The TPM mass concentrations of coking furnaces A, B, C, and D were 130 ± 11.1, 84.4 ± 6.36, 35.1 ± 17.0, and 63.8 ± 13.0 mg/m^3^, respectively. The main component of TPM was CPM, which accounted for 98%, 95%, 68%, and 95% of the mass concentrations of furnaces A, B, C, and D, respectively. Hence, the control of CPM emitted by stationary sources was an important measure for reducing atmospheric PM_2.5_ and its precursors toward improving the air quality in China.
2. Tests of water-soluble ions and elements in inorganic CPM showed that water-soluble ions were important components of CPM, and the total CPM concentration of furnaces A, B, C, and D accounted for 70%, 87%, 42%, and 66%, respectively. The characteristics of CPM were related to the flue-gas treatment technology used and the operation level of the coking facilities. Compared with the treatment process “SCR denitrification + limestone–gypsum wet desulphurization + spraying NaOH solution” employed in furnaces A and B, the treatment process “activated carbon flue-gas countercurrent-integrated purification technology + ammonia injection” employed in furnaces C and D had little effect on the removal of CPM. The total concentrations of the elements in inorganic CPM emitted by furnaces A, B, C, and D were 74.6 ± 5.31, 63.0 ± 12.3, 10.5 ± 12.2, and 7.34 ± 4.12 µg/m^3^, respectively. Ba and Al in CPM emitted by furnaces A and B were considerably high, which accounted for 91% of the total elemental mass concentrations. The toxic and harmful heavy metals detected in them included Mn, Cr, Ni, Cu, Zn, As, Cd, and Pb.
3. Methanol, acetone, and n-hexane were selected as the final constant volume solvents, and the full-scan ion current diagram was obtained via GC–MS. Based on the results of analyzing the full-scan ion flow graph obtained by three solvents, there were many types of organic compounds in CPM. The area normalization method was used to obtain the 15 main organic components in CPM. The proportion of n-alkanes was the highest, followed by those of esters and phenols. Most of these organic substances in the atmosphere were toxic to humans and ecosystems.
4. From the perspective of source and sink dynamics, studies on the monitoring of CPM emissions containing metals, especially heavy metals, from fixed sources and the pollution caused by emissions and the ecological effects of pollution would provide a scientific basis for guaranteeing the safety of the ecological environment. Because of the particular formation mechanism of CPM and the condensation process of PM_2.5_ in the atmosphere, the flue-gas treatment technology currently being used in China should be optimized, and the collaborative technologies that could be used to treat particulate matter and other pollutants based on condensation mechanisms should be developed. The use of new nanofiltration materials for removing PM_2.5_ was effective, and it would play an important role in regional air pollution control as well as in improving human health and ecosystems.

Although this study reported the emission status of FPM, CPM, and TPM in flue gas emitted by coking plants with typical flue-gas treatment technology in northern Henan of China as well as analyzed the formation mechanism of the main components of CPM in combination with the flue-gas treatment process, the number of emission sources measured was still small due to the limitation of the monitoring conditions. To fully grasp the CPM status in flue gas emitted by Chinese coking plants under the existing flue-gas treatment conditions, more emission source monitoring is required. Moreover, the flue-gas treatment process with the best TPM treatment effect should be selected and developed to provide a technical basis for the emission reduction of PM_2.5_ and its preconditions in the atmosphere.

## Data Availability

All data generated or analyzed during this study are included in this published article.
